# Carbon Materials Derived from Poly(aniline-*co*-*p*-phenylenediamine) Cryogels

**DOI:** 10.3390/polym12010011

**Published:** 2019-12-19

**Authors:** Konstantin A. Milakin, Nemanja Gavrilov, Igor A. Pašti, Miroslava Trchová, Beata A. Zasońska, Jaroslav Stejskal, Patrycja Bober

**Affiliations:** 1 Institute of Macromolecular Chemistry, Academy of Sciences of the Czech Republic, Heyrovsky Sq. 2, 162 06 Prague 6, Czech Republic; milakin@imc.cas.cz (K.A.M.); trchova@imc.cas.cz (M.T.); zasonska@imc.cas.cz (B.A.Z.); stejskal@imc.cas.cz (J.S.); 2 Faculty of Physical Chemistry, University of Belgrade, Studentski trg 12–16, 11158 Belgrade, Serbia; gavrilov@ffh.bg.ac.rs (N.G.); igor@ffh.bg.ac.rs (I.A.P.)

**Keywords:** poly(aniline-*co*-*p*-phenylenediamine), cryogels, carbonization, specific surface area, capacitance

## Abstract

Nitrogen-containing carbon derivatives were prepared by the carbonization of poly(aniline-*co*-*p*-phenylenediamine) cryogels in inert atmosphere. Lower aniline fraction in the comonomer mixture used for preparation of the cryogels led to the decrease of their thermal stability, a consequent increase of carbonization degree, and less defective structure of carbonized materials. The resulting carbonaceous products had up to 4 orders of magnitude higher specific surface area than their respective cryogel precursors, the highest value 931 m^2^ g^−1^ being achieved for carbonized poly(*p*-phenylenediamine) cryogel. Electrochemical characterization of the carbon derivatives demonstrated that the decrease in aniline concentration during the synthesis of the precursor cryogels led to higher gravimetric capacitance for corresponding carbonized materials. These materials can potentially be used for energy storage applications.

## 1. Introduction

Nitrogen-containing carbon materials have attracted considerable attention due to promising electronic and physicochemical properties [1] which determine their potential applications as electrode materials for fuel cells and batteries [2,3,4], sensors [5,6,7], catalysts [8], electrocatalysts [5], supercapacitors [9,10,11], and adsorbents [9,12,13,14,15]. Nitrogen-enriched carbon derivatives can be obtained by a variety of flexible methods, which include chemical vapor deposition [2,6,16,17], arc discharge [18,19], thermal treatment of C/N-containing compounds or mixtures in inert atmosphere [3,7,8,10,12] and annealing of materials in inert atmosphere [5,14,15]. Polyaniline and polyaniline-based materials containing both carbon and nitrogen atoms represent convenient precursors for the preparation of nitrogen-containing carbon derivatives. Their morphology varying from globules to nanotubes [20] and macroporous structures [21] is preserved after carbonization [22,23,24] and can be reliably set during the initial synthesis. Nitrogen content in the materials can also be controlled by chemical modification of the polymer structure, for instance by the copolymerization approach [25].

The preparation of novel nitrogen-containing carbon materials by the carbonization of freeze-dried macroporous polyaniline/poly(vinyl alcohol) cryogels has recently been reported [24]. It was shown that even after decomposition of poly(vinyl alcohol), which served as a mechanical support in the initial cryogels, the resulting carbogels retained the macroporous structure of their precursors, while having 50 times higher specific surface area. This was achieved in a single carbonization step without additional activation. Due to its high surface area, this type of material can potentially be useful for application as an electrochemical capacitor. However, for designing novel electrode materials, it would be beneficial to control their nitrogen content, which can directly influence their performance [16]. The copolymerization of aniline with *p*-phenylenediamine can be a convenient way how to achieve this goal by offering a facile way to control a composition of a precursor polymer [26,27]. The incorporation of *p*-phenylenediamine constitutional units to the polymer structure not only allows the variation of the nitrogen-to-carbon ratio, but it might also influence specific surface area of the carbonized material according to the difference in values obtained for carbonized polyaniline and poly(*p*-phenylenediamine), 14 [28] and 259 m^2^ g^−1^ [29], respectively. Thus, in the present paper, we have prepared carbons by the carbonization of recently reported poly(aniline-*co*-*p*-phenylenediamine)/poly(vinyl alcohol) (poly(ANI-*co*-PPDA)/PVAL) cryogels [30] and studied the influence of the precursor composition on the structure and properties of resulting materials.

## 2. Experimental

### 2.1. Preparation of Cryogels

Aniline hydrochloride (Penta, Prague, Czech Republic), *p*-phenylenediamine dihydrochloride (Sigma-Aldrich, Saint Louis, MO, USA), ammonium peroxydisulfate (Lach-Ner, Neratovice, Czech Republic) and poly(vinyl alcohol) (Mowiol 10−98, molecular weight 61,000, Sigma-Aldrich, Saint Louis, MO, USA) were used as received.

Poly(aniline-*co*-*p*-phenylenediamine)/poly(vinyl alcohol) cryogels were prepared by the oxidative copolymerization as described previously [30]. A pre-cooled (−4 °C) solution containing aniline hydrochloride and *p*-phenylenediamine dihydrochloride (various comonomer mole ratios, total molar concentration 0.8 M) in 5 wt% aqueous poly(vinyl alcohol) was mixed with equal volume of pre-cooled 1 M ammonium peroxydisulfate solution also in 5 wt% aqueous poly(vinyl alcohol). The resulting mixture was drawn into polyethylene syringes, frozen in solid CO_2_/ethanol suspension at −78 °C, and left to polymerize in a freezer at −24 °C for 7 days. After the polymerization and thawing of the cryogels at room temperature, these were removed from the syringes, exhaustively washed with water to remove reaction by-products, and freeze dried ((GREGOR Instruments L4-110 freeze-drier paired with Vacuubrand RC 6 pump, Říčany, Czech Republic).

### 2.2. Carbonization

The freeze-dried cryogels were carbonized in nitrogen atmosphere by heating up to 650 °C in an electric oven at a rate of 15 °C min^−1^, and subsequent passive cooling down for approximately 8 h. The carbonization yield was determined as a ratio of the sample mass after the carbonization to the mass of the initial sample.

### 2.3. Characterization

Thermogravimetric analysis (TGA) was performed using a Perkin Elmer Pyris 1 Thermogravimetric Analyzer (Perkin Elmer, Waltham, MA, USA) in a temperature range 35–850 °C at a heating rate of 10 °C min^−1^. Air or nitrogen flow-rate was fixed at 25 mL min^−1^. The morphology of carbonized cryogels was investigated by a scanning electron microscope (SEM) MAIA3 Tescan (Tescan, Brno, Czech Republic).

The Brunauer–Emmett–Teller (BET) specific surface area of the samples was determined using a Gemini VII 2390a (Micromeritics, Instruments Corp, Norcross, GA, USA) with nitrogen as the sorbate. The samples were vacuum dried at room temperature, and the carbonized samples at 60 °C for 50 h. Contents of carbon and nitrogen were determined on a Perkin-Elmer 2400 CHN elemental analyzer (Waltham, MA, USA).

Raman spectra were recorded with a Renishaw inVia Reflex Raman microspectrometer after excitation with a HeNe 633 nm laser. A research-grade Leica DM LM microscope (Leica Microsystems, Wetzlar, Germany) was used to focus the laser beam. The scattered light was analyzed with a spectrograph using holographic grating 1800 lines mm^−1^. A Peltier-cooled charge-coupled device (CCD) detector (576 × 384 pixels) registered the dispersed light.

Thin film electrodes were prepared by drop casting of carbon ink, as described in the previous paper [31]. Briefly, 5 mg of desired material was dispersed in a 400 μL ethanol/590 μL deionized water/10 µL of 0.5 wt% Nafion^®^ solution in ethanol, and homogenized via ultrasound for 30 min. Films were formed by evaporation of solvent under a mild nitrogen gas stream from 10 µL droplet of the carbon ink adhered to the glassy carbon (GC) disk electrode having geometric surface area 0.196 cm^2^. Specific mass loading was 250 µg cm^−2^, which was further used in the calculation of material capacitance. Electrochemical tests were done in a standard three-electrode setup with a high surface area platinum as a counter electrode and a reference saturated calomel electrode (SCE). Cyclic voltammetry (CV) was used to assess the capacitance of the synthetized materials in a 3 M aqueous KOH with an Ivium VO1107 potentiostat/galvanostat (Ivium Technologies B.V., Eindhoven, The Netherlands). Gravimetric capacitances (*C*, in F g^−1^) were calculated using the following formula:

(1) $$C=\frac{Q}{2\cdot \Delta V\cdot m}=\frac{\int idt}{2\cdot \Delta V\cdot m}$$

where *Q* is the charge passed, obtained by integration of positive and negative sweep in cyclic voltammograms, Δ*V* is the explored potential window and *m* is the mass of the active material deposited on the surface of GC. Galvanostatic charge/discharge (GCD) was also performed using different current loads to compare with the results of cyclic voltammetry in the same three-electrode system. The electrolyte was purged with nitrogen for 15 min prior to the experiment and a mild flow was kept just underneath the electrolyte surface during measurement. Measurements were done at room temperature, 25.0 ± 0.5 °C.

## 3. Results and Discussion

The copolymerization of aniline and *p*-phenylenediamine by ammonium peroxydisulfate in the frozen poly(vinyl alcohol) solution led to the formation of macroporous poly(ANI-*co*-PPDA)/PVAL cryogels, which were earlier characterized in detail [30]. In order to proceed with the carbonization of cryogels, it was necessary to study at first their thermal stability in air and nitrogen atmosphere by TGA.

### 3.1. Thermogravimetric Analysis

**Figure 1:**
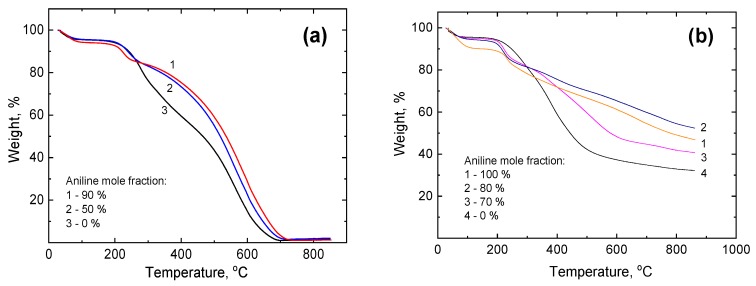
Thermogravimetric curves of poly(ANI-co-PPDA)/PVAL cryogels prepared using various aniline fractions recorded (**a**) in air and (**b**) in inert atmosphere.

TGA curves of poly(ANI-*co*-PPDA)/PVAL copolymer cryogels with different composition recorded in air are given in Figure 1a. A typical TGA curve of a poly(ANI-*co*-PPDA)/PVAL cryogel contains three main weight loss regions. For example, for PANI cryogel prepared using 90 mol% of aniline they are as follows: (1) up to 120 °C corresponding to loss of water, (2) around 200–280 °C—decomposition of poly(vinyl alcohol) and loss of the acid protonating polyaniline or poly(*p*-phenylenediamine), and (3) around 440–730 °C—decomposition of poly(vinyl alcohol) along with conducting polymer phase [32,33,34]. Analysis of the thermal behavior of cryogels prepared with various comonomer ratios show that the decrease in aniline fraction in the sample reduces cryogel stability in the temperature region from 300 to 700 °C. In this case, *p*-phenylenediamine acts as a less thermally stable component in the cryogels.

Thermogravimetric curves of poly(ANI-*co*-PPDA)/PVAL cryogels (Figure 1b), having various aniline fractions recorded in nitrogen atmosphere, demonstrate that the total weight loss of samples during the analysis is, as expected, much lower in the comparison to the TGA in air, where the samples were decomposed completely. The residual weight at 860 °C for the cryogels varies in the region of about 30–50 wt%. After taking into account mass loss attributed to water evolution from the cryogels, which occurs in the temperature region below 120 °C [35], it can be concluded that, similarly to TGA in air, the thermal stability of poly(ANI-*co*-PPDA)/PVAL cryogels under inert conditions also decreases with the decrease of aniline fraction in initial reaction mixture. Therefore, thermal treatment of poly(ANI-*co*-PPDA)/PVAL cryogels in inert atmosphere can be used for preparation of carbon-based materials with a reasonable yield. According to the thermal stability trend, a higher carbonization yield can be expected for cryogels enriched with *p*-phenylenediamine units, but not for the poly(*p*-phenylenediamine) alone.

### 3.2. Carbonization Yield

Poly(ANI-*co*-PPDA)/PVAL-C carbonized materials were obtained in a preparative way by heating respective poly(ANI-*co*-PPDA)/PVAL cryogels up to 650 °C in nitrogen atmosphere. The carbonization temperature was chosen based on the previous research [36], where it was shown to be the minimum temperature which led to complete carbonization of polyaniline still in good yield. Table 1 shows carbonization yields of poly(ANI-*co*-PPDA)/PVAL cryogels containing different aniline fractions. The yields varied in the region of 34%–45%, which correlated with the results obtained by TGA in nitrogen atmosphere. Slightly lower carbonization yield in comparison to residual mass values obtained from TGA in inert atmosphere might be explained by longer exposure of the samples to elevated temperatures during the carbonization. Elemental analysis of the prepared carbonized materials showed that all of them had N/C weight ratio from 0.11 to 0.15.

### 3.3. Raman Spectroscopy

**Figure 2:**
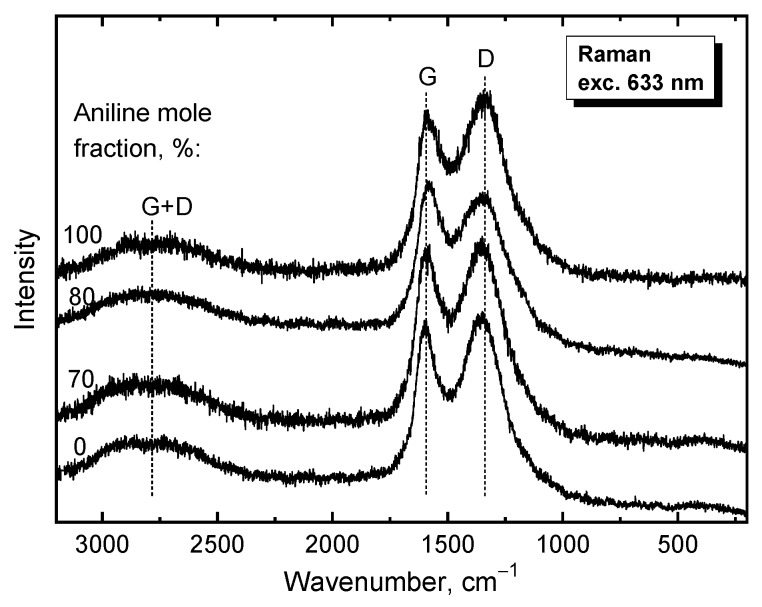
Raman spectra of carbonized poly(ANI-*co*-PPDA)/PVAL-C materials prepared from the cryogels synthesized using various aniline fractions. Excitation wavelength 633 nm.

Raman spectroscopy is a method of choice for the characterization of the carbonization. Raman spectra of carbonized poly(ANI-*co*-PPDA)/PVAL-C materials prepared from the cryogels having various aniline fractions (Figure 2) exhibit two main bands located at around 1350 cm^−1^—the disorder induced band (D band) and the 1600 cm^−1^—the graphitic band (G band) [37]. They correspond to the molecular structure of a typical disordered carbon-like material.

The degree of structural disorder is characterized by the *I*_D_/*I*_G_ ratios determined by integrated peak areas (Table 1), which were calculated from the Raman spectra (Figure 2). These values decreased with the decreasing the amount of aniline mole fraction in the initial samples, which corresponds to an increase of carbonization degree, which is in the good agreement with TGA results. The relatively high values of the *I*_D_/*I*_G_ and the broadening of the G and D bands indicate a high degree of structural disorder in these materials. This is connected with incorporation of nitrogen in the carbon sp^2^ network [37]. This statement is supported by the presence of a G + D combination band at about 2750 cm^−1^ observed in the spectra in the presence of defects (Figure 2).

### 3.4. Morphology

**Figure 3:**
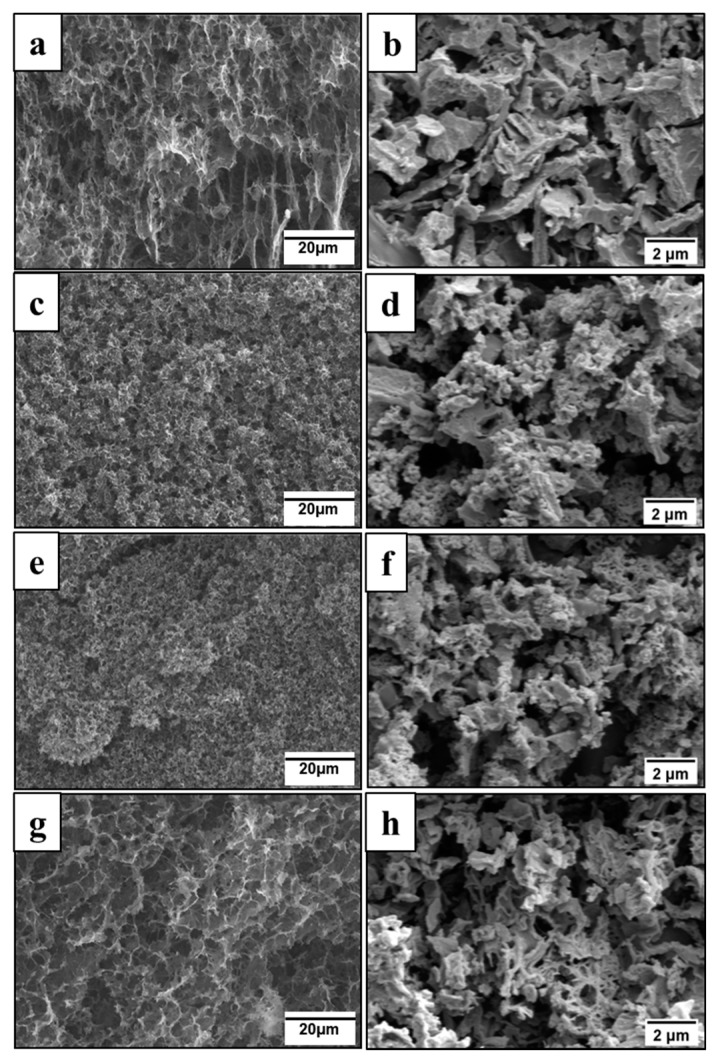
Scanning electron microscopy images of initial (**a**,**c**,**e**,**g**) and carbonized (**b**,**d**,**f**,**h**) poly(ANI-*co*-PPDA)/PVAL cryogels prepared using various aniline fractions: (**a**,**b**) 100 mol%, (**c**,**d**) 80 mol%, (**e**,**f**) 70 mol%, and (**g**,**h**) 0 mol%.

As shown before [30], poly(ANI-*co*-PPDA)/PVAL cryogels have a macroporous structure with a pore size up to tens of micrometers, which is dependent on copolymer composition (Figure 3a,c,e,g). Evolution of the materials morphology after carbonization was assessed by SEM (Figure 3b,d,f,h).

The macroporous structure of initial cryogels was not preserved after carbonization (Figure 3), unlike the previously reported result where the morphology of the carbogels produced from poly(ANI)/PVAL cryogels was found to be retained [24]. Carbonized materials in general show significantly poorer mechanical stability due to complete pyrolysis of PVAL under experimental conditions [38], which served as a mechanical support in initial cryogels and shrinking of the gels during the carbonization. The principal difference from the previous work [24] might be explained by a possibly different pore structure of cryogels induced by a higher concentration of the monomer used and a different monomer/poly(vinyl alcohol) ratio, which might influence the distribution of a produced polymer across the cryogel volume. Resulting carbonized materials have different morphology depending on their composition. With the decrease of aniline fraction in the cryogel, the carbonized cryogels changed from agglomerates of complex morphology for carbonized polyaniline homopolymer cryogels to sponge-like particles with visible submicrometer-sized pores for carbonized poly(*p*-phenylenediamine) homopolymer cryogels.

### 3.5. Specific Surface Area

Specific surface area and pore volume of carbonized poly(ANI-*co*-PPDA)/PVAL-C cryogels, which are important parameters for potential electrochemical applications of the prepared materials, were assessed by a nitrogen-absorption technique. The carbonization of the cryogels led to the increase of their specific surface area by one order of magnitude for the cryogels prepared using 100, 80 and 70 mol% of aniline and by 4 orders of magnitude for poly(*p*-phenylenediamine) cryogel (Table 2), despite the fact that all carbonized materials did not keep their macroporous structure after carbonization process. Analysis of the specific surface area for the carbonized cryogels also shows that this parameter decreases with the decrease of aniline fraction in monomer mixture. This is in good agreement with SEM results and changes in the morphology of carbonized materials. It should be especially noted that polyaniline and poly(*p*-phenylenediamine) powders carbonized under the similar conditions showed much lower specific surface areas, 14 [28] and 259 m^2^ g^−1^ [29], respectively, therefore using poly(ANI-*co*-PPDA)/PVAL cryogels as a precursor for a carbonized material achieves higher values. This might be of benefit for electrode materials in supercapacitors [27].

### 3.6. Electrochemistry

**Figure 4:**
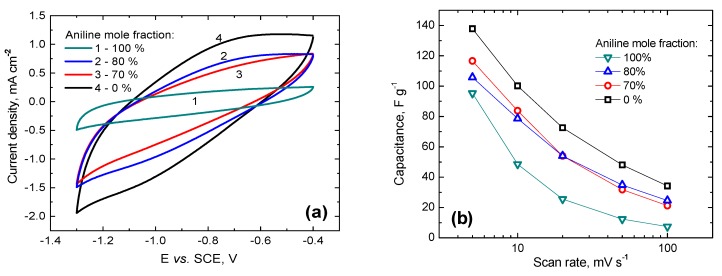
(**a**) Cyclic voltammograms of carbonized poly(ANI-*co*-PPDA)/PVAL-C cryogels recorded in quiescent N_2_-purged 3 M KOH solution at the scan rate of 100 mV s^−1^ and (**b**) gravimetric capacitance measured at different scan rates.

Electrochemical characterization of carbonized poly(ANI-*co*-PPDA)/PVAL-C cryogels was performed by cyclic voltammetry and comparison of their capacitive responses in 3 M KOH at 100 mV s^−1^ sweep rate are presented in Figure 4.

From the area encompassed in Figure 4a by the forward/backward scans, it is evident that poly(*p*-phenylenediamine)-derived carbon delivers the highest capacitance. Voltammograms of materials prepared from precursors synthesized with 70 and 80 mol% of aniline have a somewhat smaller area. Quantification is given in Table 2. The shape of all three cyclic voltammetric curves recorded for the carbonized *p*-phenylenediamine-containing materials (Figure 4a; 0, 70 and 80%) display similar features; namely, an inclined rectangle which indicates a significant resistance in explored samples and a “hump” centered around −0.7 V in the forward going scan and at −1.1 V in the negative sweep, probably due to pseudo-faradaic processes ascribed to electrochemical transformations of nitrogen groups produced from poly(*p*-phenylenediamine) upon carbonization. By contrast, the carbonized material prepared from polyaniline cryogel (Figure 4a; 100%) delivers significantly smaller capacitance with a voltammogram that is almost horizontal indicating lower resistance and absence of pseudo-faradaic maxima.

**Figure 5:**
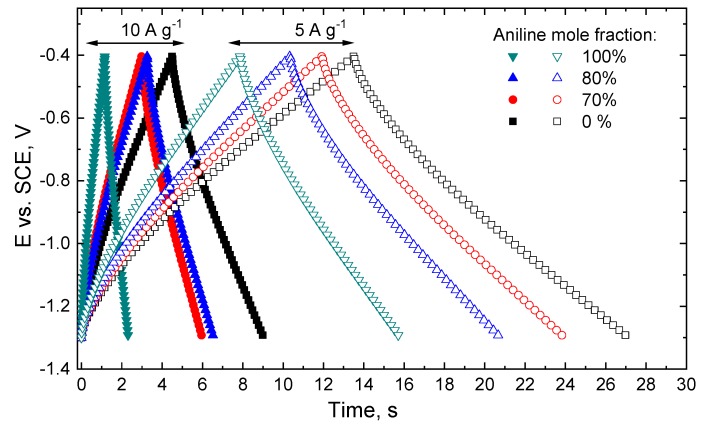
Galvanostatic charge/discharge tests of carbonized poly(ANI-*co*-PPDA)/PVAL-C cryogels at the current loads of 5 and 10 A g^−1^ recorded in quiescent N_2_-purged 3 M KOH solution.

The results obtained for cyclic voltammetry are in agreement with the ones obtained by GCD, showing the same trend in terms of capacitive response in the series of investigated materials (Figure 5). Recorded GCD curves point to large capacitance fade with increasing current load, consistent with cyclic voltammetry, but the curves are generally featureless and preserve the shape with an increasing current load.

All tested carbonized poly(ANI-*co*-PPDA)/PVAL-C cryogels are sensitive to the increasing potential sweep rate with significant capacitance fade (Figure 4b). This huge drop in delivered capacitance is a trait of carbon materials that lack sufficient conductivity, which might be circumvented by the addition of some conduction-enhancing additive. Capacitive response correlates to some extent to the specific surface area, with the highest values delivered by the material with the largest pore volume and the highest surface area. To try to elucidate the contribution from electric double-layer capacitance and pseudo-capacitance, we conducted a Dunn analysis [39] on the carbonized poly(*p*-phenylenediamine) as it delivered the highest capacitance among the probed materials. Figure S1 in Supplementary Materials reveals that the contribution from pseudo-capacitance in poly(*p*-phenylenediamine)-derived carbon is limited to a few percent, reiterating the dominant role of the surface area on the capacitance. This result is in fact surprising, considering that a large contribution of double layer capacitance should allow for very fast charging at any potential sweep rate/current load. However, if one evaluates expected capacitance of the materials, taking typical 20 μF cm^−2^ as a real capacitance, it is clear that carbonized poly(*p*-phenylenediamine) does not have all of its specific surface available to the electrolyte. For this reason, we believe that a poor rate capability of investigated materials is due to limited access of the electrolyte to the pores in the interior of materials and hindered diffusion at high potential sweep rates or current loads. In other words, during fast scans, a significant amount of electrolyte gets trapped in the pores and cannot respond to rapid potential modulations, resulting in low capacitances.

## 4. Conclusions

Nitrogen-containing carbon derivatives were prepared by one-step carbonization of freeze-dried poly(ANI-*co*-PPDA)/PVAL cryogels. Although macroporous cryogel morphology was not preserved upon carbonization, the specific surface area of the resulting materials was found to increase by up to 4 orders of magnitude. It reached the highest value 931 m^2^ g^-1^ for carbonized poly(*p*-phenylenediamine) cryogel, which is larger than that for its carbonized powder analogue. In addition to increasing specific surface area, lower aniline fraction used for the preparation of the precursor cryogels, and their corresponding lower thermal stability led to production of carbon derivatives with fewer defects. These carbonized copolymer materials can potentially be used for electrochemical energy-storage applications, and their gravimetric capacitance was found to be enhanced with a decrease of aniline content in the synthesis of precursor cryogels.

## Figures and Tables

**Table 1:** Carbonization yield of poly(ANI-*co*-PPDA)/PVAL cryogels prepared using different aniline fractions. *I*_D_/*I_G_* are ratios of integrated surface area in the Raman spectra of carbonized poly(ANI-*co*-PPDA)/PVAL-C materials.

Aniline Mole Fraction, %	Carbonization Yield, %	*I*_D_/*I*_G_
100 (PANI)	39	2.96
80	45	2.88
70	35	2.51
0 (PPDA)	34	2.39

**Table 2:** Specific surface area, pore volume of poly(ANI-*co*-PPDA)/PVAL cryogels prepared at various aniline fractions before (Data from reference [30]) and after carbonization, and gravimetric capacitance of the carbonized materials evaluated at different scan rates.

Aniline Mole Fraction, %	Before Carbonization		After Carbonization			
	Specific Surface Area, m^2^·g^−1^	Pore Volume, cm^3^·g^−1^	Specific Surface Area, m^2^·g^−1^	Pore Volume, cm³·g^−1^	Capacitance at 5 mV·s^−1^, F·g^−1^	Capacitance at 100 mV·s^−1^, F·g^−1^
100	18	0.016	328	0.34	102	7.4
80	75	0.020	325	0.11	106	24.6
70	78	0.012	511	0.24	117	21.2
0	0.11	0.014	931	0.42	138	34.2

## Supplementary Materials

Are available online at https://www.mdpi.com/2073-4360/12/1/11/s1, Figure S1: Cyclic voltammogram of carbonized poly(*p*-phenylenediamine) derived carbon recorded in quiescent nitrogen-purged 3 M KOH solution at the scan rate of 10 mV s^−1^ and an electric double-layer capacitance contribution evaluated by Dunn method.
