# Supplementary material for: Carbon Materials Derived from Poly(aniline-co-p-phenylenediamine) Cryogels
Source: Polymers (Basel). 2019 Dec 19;12(1):11. doi: 10.3390/polym12010011 (PMC7022277; doi:10.3390/polym12010011)
Supplement: Supplementary file 1 [file polymers-12-00011-s001.pdf]

Supporting Information for:

Carbon Materials Derived from Poly(aniline-*co*-*p*-phenylenediamine) Cryogels

Konstantin A. Milakin [1], Nemanja Gavrilov [2], Igor A. Pašti [2], Miroslava Trchová [1], Beata A. Zasońska [1], Jaroslav Stejskal [1] and Patrycja Bober [1,*]

[1] Institute of Macromolecular Chemistry, Academy of Sciences of the Czech Republic, Heyrovsky Sq. 2, 162 06 Prague 6, Czech Republic

[2] Faculty of Physical Chemistry, University of Belgrade, Studentski trg 12–16, 11158 Belgrade, Serbia

* Correspondence: bober@imc.cas.cz; Tel.: +420-296-809-443

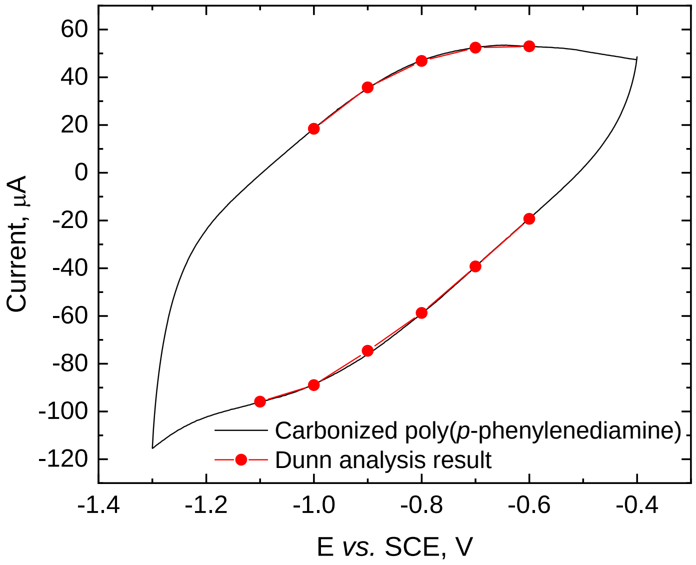

Figure S1. Cyclic voltammogram of carbonized poly(*p*-phenylenediamine) derived carbon recorded in quiescent nitrogen-purged 3 M KOH solution at the scan rate of 10 mV s^{-1} and an electric double layer capacitance contribution evaluated by Dunn method.
